# Antimicrobial activity of the volatile substances from essential oils

**DOI:** 10.1186/s12906-021-03285-3

**Published:** 2021-04-17

**Authors:** Mareshah Abers, Sydney Schroeder, Linna Goelz, Adrienne Sulser, Tiffany St. Rose, Keely Puchalski, Jeffrey Langland

**Affiliations:** Southwest College of Naturopathic Medicine, The Ric Scalzo Institute for Botanical Research, Tempe, AZ 85282 USA

**Keywords:** Essential oils, Volatile substances, Antimicrobials

## Abstract

**Background:**

Essential oils are volatile and lipophilic liquid extracts made from plants as secondary metabolites that can be obtained by distillation. To date, several studies have investigated the *direct* antimicrobial activity of liquid essential oils. However, this study investigated the antimicrobial properties of the *volatile substances* present in various essential oils.

**Methods:**

A modified zone of inhibition protocol was developed using agar petri dishes with a center glass vial to allow evaporation and aerosolization of the potential active constituents from essential oils. In total, nineteen essential oils were tested against five Gram positive bacterial species, five Gram negative bacterial species and one fungi.

**Results:**

This study found potent antimicrobial activity from the volatile constituents of several essential oils. Rosemary, tea tree, and cassia volatiles were found to be the best broad-spectrum antibacterial agents, whereas clove volatiles had almost no antimicrobial activity.

**Conclusions:**

These results support the anecdotal historical evidence of the antimicrobial activity of the volatile constituents essential oils. Modern medical implications for this work may be related to the use of aromatic essential oils for respiratory or dermatological infections.

**Supplementary Information:**

The online version contains supplementary material available at 10.1186/s12906-021-03285-3.

## Background

Infectious disease has plagued humanity since ancient times [1]. In 1923, Alexander Fleming gave a glimmer of hope in eradicating infectious disease when he discovered penicillin [2]. This began the modern war against bacterial pathogens, but since this discovery, the unregulated usage and the over-prescription of antibiotics has increased the prevalence of antibiotic resistant bacteria [3]. Advances in medicine have led to the exploration of novel therapies to reduce the threat of these antibiotic resistant pathogens. Essential oils may provide such resource associated with their historical applications as strong antimicrobial agents [4–9].

Essential oils are volatile, lipophilic liquid extracts mainly obtained from plants by distillation [5, 10–12]. The constituents present in essential oils are produced as secondary metabolites by plants to help their survival against environmental stressors, including pathogens. The use of essential oils dates back to Ancient Egypt where they extracted by steeping plant parts into animal fats and vegetable oils [13]. Around 1000 AD, a turning point for botanical extraction came when Avicenna from Arabia invented the steam distillation method which is now the industry standard for extracting most essential oils [14]. Beginning in 1347, the *Yersinia pestis* outbreak known as “The Great Plague” became widespread and was responsible for killing one-third of the European population [15]. It was said that people exposed to essential oils were ‘immune’ to deleterious effects of this bacteria [16]. In 1937, Rene Gattefosse, a French chemist, coined the term “Aromatherapy” and began research in essential oils that demonstrated their therapeutic properties [6, 17]. Today, essential oils are used to treat a wide array of medical conditions including cancer, pain, stress, and infectious disease [6, 12, 17, 18].

The therapeutic application of essential oils is accomplished aromatically, topically, or internally. The vast majority of essential oil research has focused on the antimicrobial activity through *direct contact* with the liquid form of the oil [19]. Only limited research is available on the antimicrobial activity of the *airborne evaporative* volatile constituents of essential oils. In the mid-1900’s, Maruzzella and Kienholz were the first researchers who used a modified antimicrobial disk diffusion assay with essential oils using saturated disks on inverted petri dish lids [4, 20]. Their research supported the antimicrobial activity of essential oil volatile constituents, but this method did not allow for quantitative comparison between essential oils. Further research found that using specific amounts of essential oils to impregnant filter disks did not change the observed effects [18].

The development of antimicrobial therapies administered using evaporative-based applications of essential oils could aide in the fight against specific microbial infections. This study focused on evaluating and comparing the antimicrobial activity of essential oil volatile substances by exposing microbes to these airborne constituents while eliminating the usage of adulterants commonly added during other studies [5, 7, 22–27]. This study provides novel insight into the prospective use of essential oil volatile constituents to combat microbial infections and expands on the current state of knowledge on the potential efficacy of essential oils.

## Methods

### Essential oils

Nineteen essential oils were tested for their antimicrobial activity. The essential oils used were: *Melaleuca alternifolia* (Tea Tree), *Origanum vulgare* (Oregano), *Eugenia caryophyllata* (Clove), *Rosmarinus officinalis* (Rosemary), *Lavandula angustifolia* (Lavender), *Cinnamomum zeylanicum* (Cinnamon), *Boswellia carterii, sacra, papyrifera*, and *frereana* (Frankincense), *Citrus limon* (Lemon), *Thymus vulgaris* (Thyme), *Mentha piperita* (Peppermint), *Abies alba* (White Fir), *Juniperus virginiana* (Cedarwood), *Thuja plicata* (Arborvitae), *Gaultheria fragrantissima* (Wintergreen), *Foeniculum vulgare* (Fennel), *Cananga odorata* (Ylang Ylang), *Cinnamomum cassia* (Cassia), *Citrus sinensis* (Wild Orange), and *Cymbopogon flexuous* (Lemon Grass). The essential oils were all obtained from dōTERRA (Pleasant Grove, Utah). Chemical composition of the oils was verified by GC-mass-spectrometry by the supplier (dōTERRA). Analysis was done using a ZB5 column (60 m length × 0.25 mm inner diameter × 0.25 μm film thickness) with a Shimadzu GCMS-QP2010 Ultra instrument under the following conditions: Carrier gas - Helium 80 psi; Temperature ramp – 2 °C per minute up to 260 °C; Split ratio - 30:1; Sample preparation - 5%w/v solution with Dichloromethane. Voucher samples of all essential oils are deposited in a repository at Southwest College of Naturopathic Medicine. Gas chromatography profiles may be accessed at http://sourcetoyou.com with their respective lot numbers found in Table 2.

### Microbial samples

Ten microbial species (obtained from Hardy Diagnostics, Santa Maria, CA) were tested for their susceptibility to the selected essential oils: *Mycobacterium smegmatis* (ATCC 14468)*, Staphylococcus epidermidis* (ATCC 12228)*, Staphylococcus aureus* (ATCC 14775)*,* methicillin-resistant *Staphylococcus aureus* (ATCC BAA-44)*, Streptococcus pyogenes* (ATCC 12344)*, Pseudomonas aeruginosa* (ATCC 35554), antibiotic-resistant *Pseudomonas aeruginosa* (ATCC 19429)*, Bordetella bronchiseptica* (ATCC 10580)*, Klebsiella pneumoniae* (ATCC 13883)*,* and *Candida albicans* (ATCC 10231)*.* Obtained lyphophilized samples were initially grown on Tryptic Soy Agar (TSA) slants at 37 °C for 24 h. These stock cultures were stored at 4 °C and transferred to a fresh TSA slant on a monthly basis.

### Antimicrobial sensitivity assay

The following experimental design allowed the tested microbes to be exposed to the volatile substances of essential oils in a closed environment without being in direct contact with the essential oil. Since this study focused on the antimicrobial activity of the volatile constituents of essential oils, standard minimal inhibitory concentration (MIC) or minimal bactericidal concentration (MBC) assays could not be performed. Instead a modified zone of inhibition assay was developed. Custom cylinders were made out of glass to prevent any contamination or change in essential oil chemical composition. These glass cylinders were designed to fit into the center of a standard agar petri dish (85 mm diameter, 12 mm height with 6 mm agar height) with the glass cylinder measuring 10 mm in diameter and 7 mm height (with a glass thickness of 1 mm). Eighteen-hour bacterial or *Candida* broth cultures grown at 37 °C in Tryptic Soy broth or Sabouraud broth, respectively, were used to inoculate Tryptic Soy Agar (TSA) or Sabouraud Dextrose Agar (SDA) petri dishes, respectively. 1 × 10 colony forming units (cfu) in 100ul media was added to the surface of the dish and spread evenly using an L-spreader. A 10 mm plug of agar was removed from the center of the petri dish and a sterile glass cylinder containing the indicated amounts of essential oil (0 μL, 10 μL, 20 μL, 40 μL, 80 μL, 160 μL) was placed in the hole in the center of the petri dish. The petri dishes were placed in a plastic container (to prevent any air flow currents from the incubator fan) and incubated for 24 h at 37 °C in an environmentally controlled incubator. After 24 h of incubation, the zone of inhibition (diameter) was measured. All experiments were done in triplicate. Control samples included dishes with no glass cylinder or the addition of glass cylinders with no essential oils. An example of this evaporative zone of inhibition assay is shown in Supplementary materials.

### Statistical analysis

**Fig. 1:**
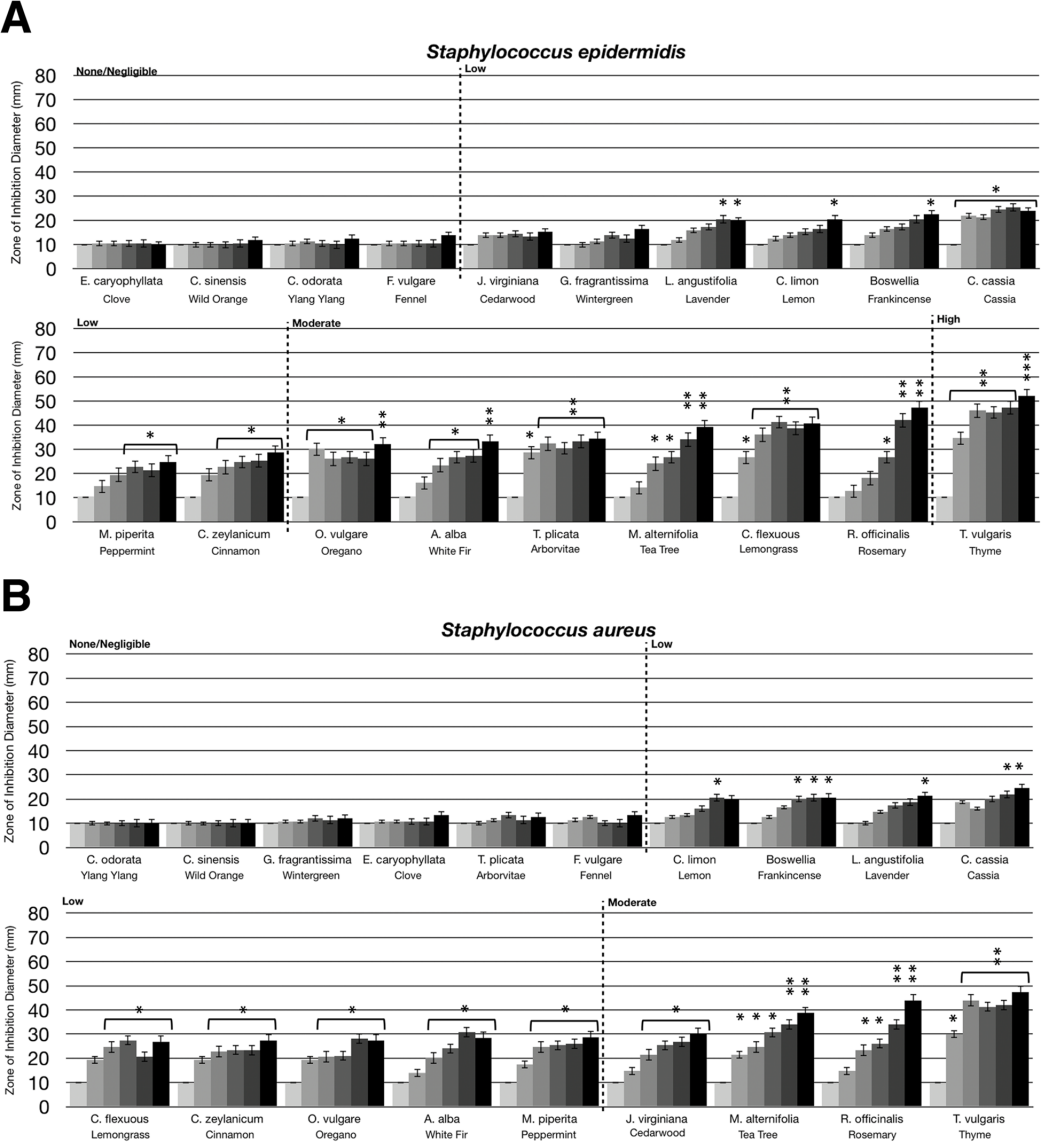

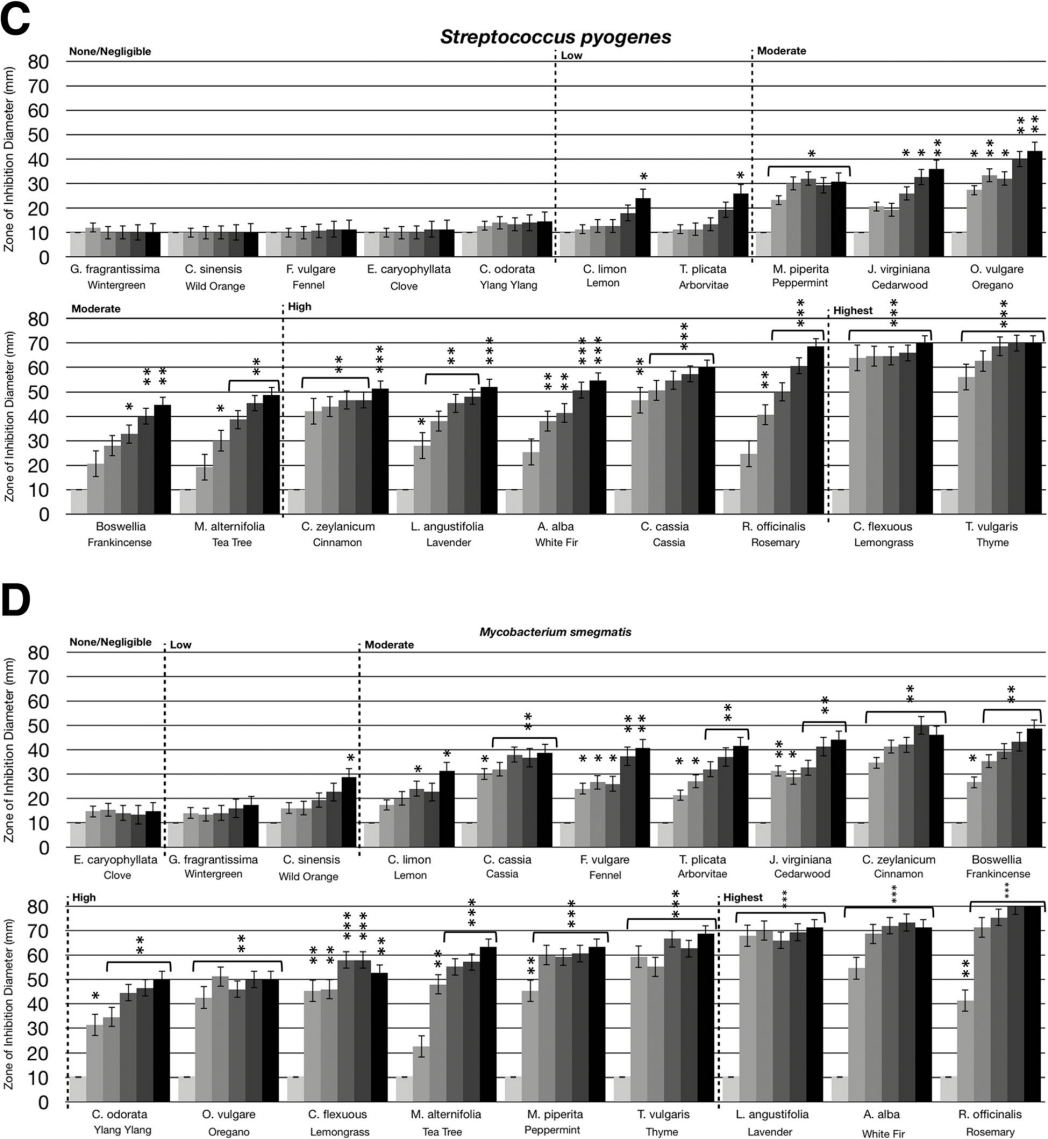
Antibacterial activity of essential oil volatile constituents against Gram positive bacteria. Eighteen-hour bacterial broth cultures (Part A: *S. epidermidis* (ATCC 12228)*,* Part B: *S. pyogenes* (ATCC 12344), Part C: *S. aureus* (ATCC 14775), Part D: *M. smegmatis* (ATCC 14468)) were used to inoculate TSA petri dishes (1 × 10 cfu/dish). A center plug of agar was removed and a sterile glass cylinder containing increasing amounts of essential oils (0 μL, 10 μL, 20 μL, 40 μL, 80 μL, 160 μL) was placed in the center of the petri dish. Petri dishes were incubated for 24 h at 37 °C. After 24 h of incubation, the zone of inhibition (diameter) was measured. The doses of 0 μL, 10 μL, 20 μL, 40 μL, 80 μL, 160 μL are shown on the graph from light grey to black, respectively. The antimicrobial activity of the essential oil volatiles was divided into six groups based on the zone of inhibition diameter: none (10 mm), negligible (10 mm - 15 mm), low (15 mm - 30 mm), moderate (30 mm - 50 mm), high (50 mm - 70 mm), and highest (70 mm - 80 mm). Error bars indicate the standard deviation from three separate trials. Statistical analysis was performed using a paired t-test. Statistically significant deviation of the various doses compared to untreated was indicated with asterisks: * *p* = 0.01–0.05; ** *p* = 0.001–0.01; ***p < 0.001

**Fig. 2:**
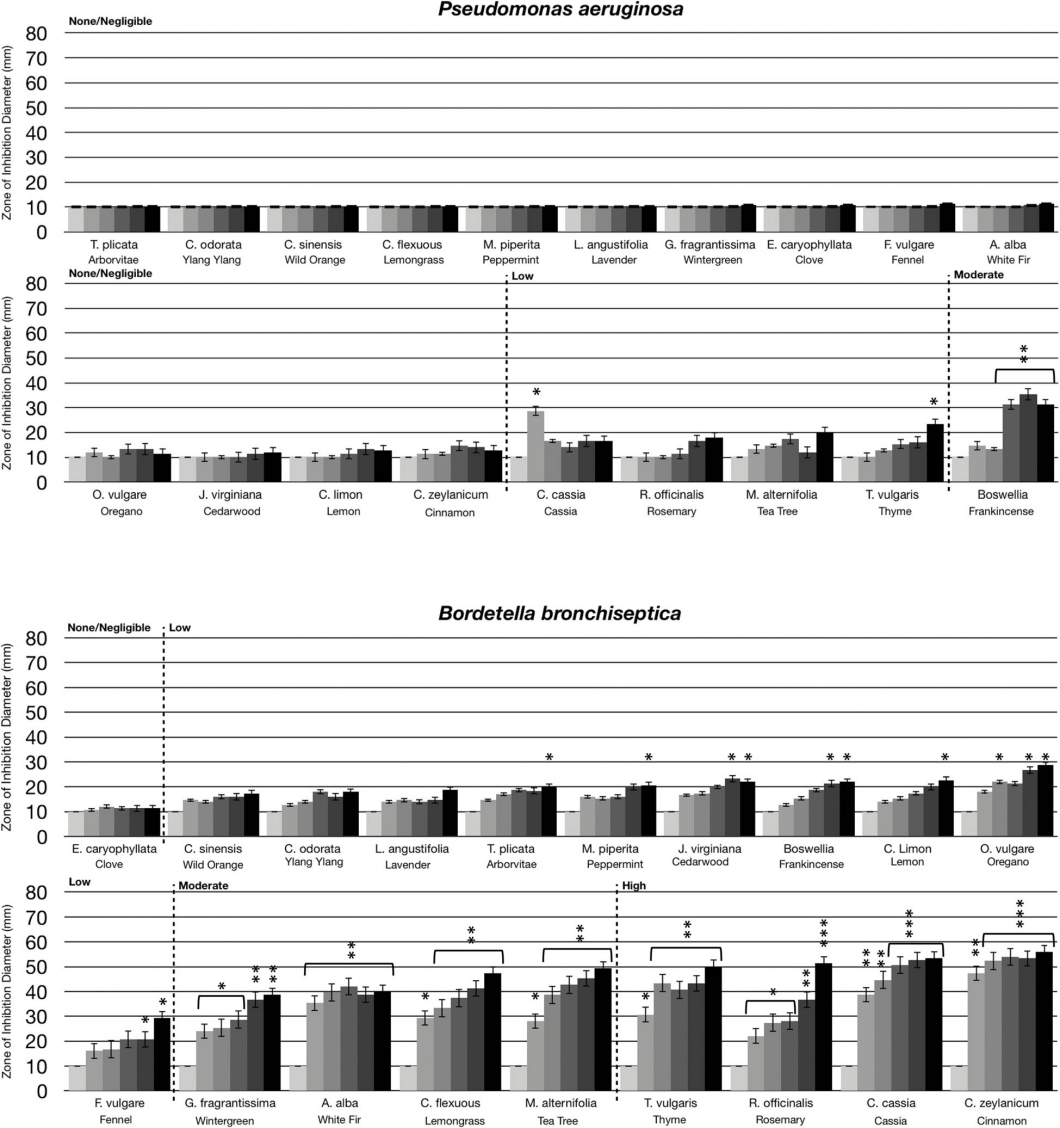

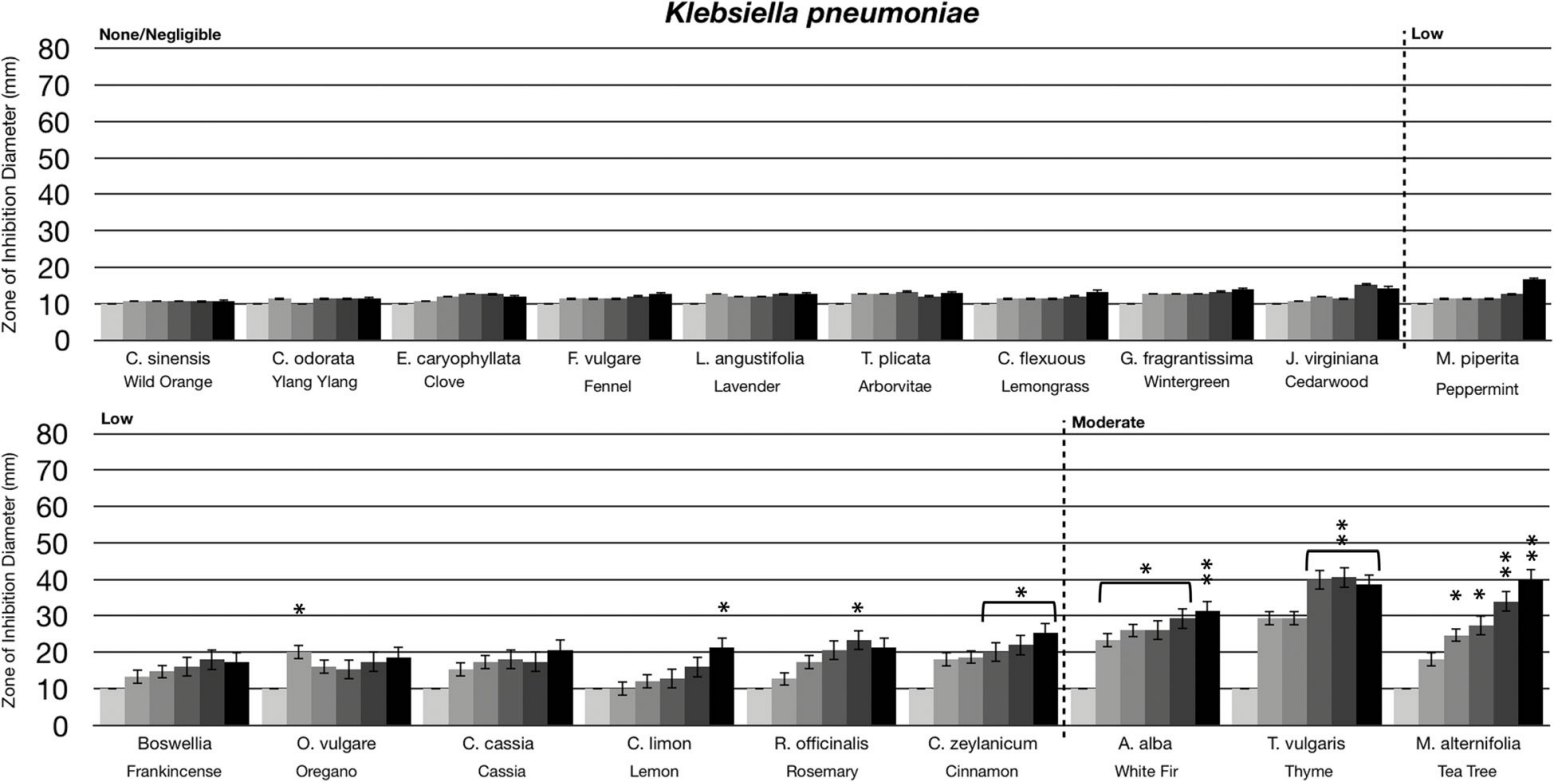
Antibacterial activity of essential oil evaporative volatile constituents against Gram negative bacteria. Eighteen-hour bacterial broth cultures (Part A: *P. aeruginosa* (ATCC 35554), Part B: *B. bronchiseptica* (ATCC 10580)*,* Part C: *K. pneumoniae* (ATCC 13883)) were used to inoculate TSA petri dishes (1 × 10 cfu/dish). A center plug of agar was removed and a sterile glass cylinder containing increasing amounts of essential oils (0 μL, 10 μL, 20 μL, 40 μL, 80 μL, 160 μL) was placed in the center of the petri dish. Petri dishes were incubated for 24 h at 37 °C. After 24 h of incubation, the zone of inhibition (diameter) was measured. The doses of 0 μL, 10 μL, 20 μL, 40 μL, 80 μL, 160 μL are shown on the graph from light grey to black, respectively. The antimicrobial activity of the essential oil volatiles was divided into six groups based on the zone of inhibition diameter: none (10 mm), negligible (10 mm - 15 mm), low (15 mm - 30 mm), moderate (30 mm - 50 mm), high (50 mm - 70 mm), and highest (70 mm - 80 mm). Error bars indicate the standard deviation from three separate trials. Statistical analysis was performed using a paired t-test. Statistically significant deviation of the various doses compared to untreated was indicated with asterisks: *p = 0.01–0.05; **p = 0.001–0.01; ***p < 0.001

**Fig. 3:**
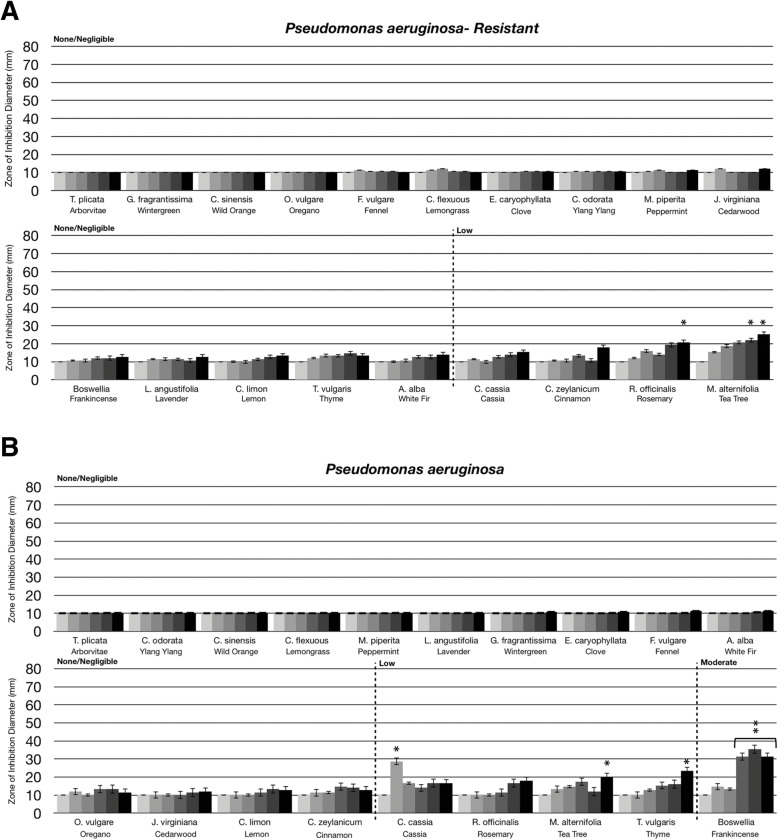

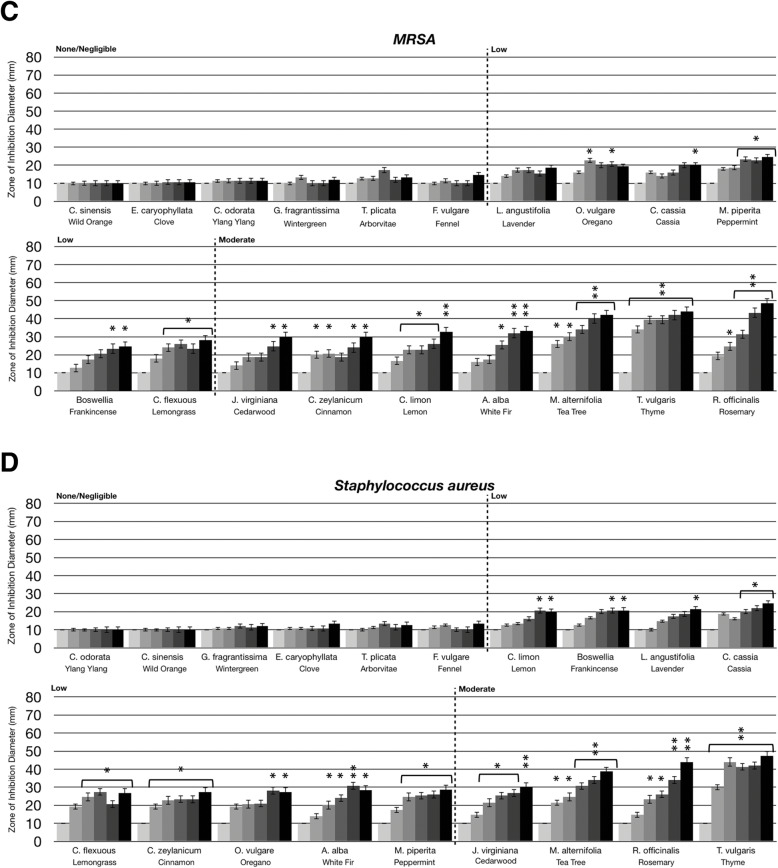
Comparison of essential oil volatile constituents antibacterial activity between antibiotic-resistant bacteria and antibiotic-sensitive bacteria. Eighteen-hour bacterial broth cultures (Part A: *Staphylococcus aureus* (ATCC 14775)*,* Part B: methicillin-resistant *Staphylococcus aureus* (ATCC BAA-44)*,* Part C: *Pseudomonas aeruginosa* (ATCC 35554), Part D: antibiotic-resistant *Pseudomonas aeruginosa* (ATCC 19429)) were used to inoculate TSA petri dishes (1 × 10 cfu/dish). A center plug of agar was removed and a sterile glass cylinder containing increasing amounts of essential oils (0 μL, 10 μL, 20 μL, 40 μL, 80 μL, 160 μL) was placed in the center of the petri dish. Petri dishes were incubated for 24 h at 37 °C. After 24 h of incubation, the zone of inhibition (diameter) was measured. The doses of 0 μL, 10 μL, 20 μL, 40 μL, 80 μL, 160 μL are shown on the graph from light grey to black, respectively. The antimicrobial activity of the essential oil volatiles was divided into six groups based on the zone of inhibition diameter: none (10 mm), negligible (10 mm - 15 mm), low (15 mm - 30 mm), moderate (30 mm - 50 mm), high (50 mm - 70 mm), and highest (70 mm - 80 mm). Error bars indicate the standard deviation from three separate trials. Statistical analysis was performed using a paired t-test. Statistically significant deviation of the various doses compared to untreated was indicated with asterisks: * *p* = 0.01–0.05; ** *p* = 0.001–0.01; ***p < 0.001

**Fig. 4:**
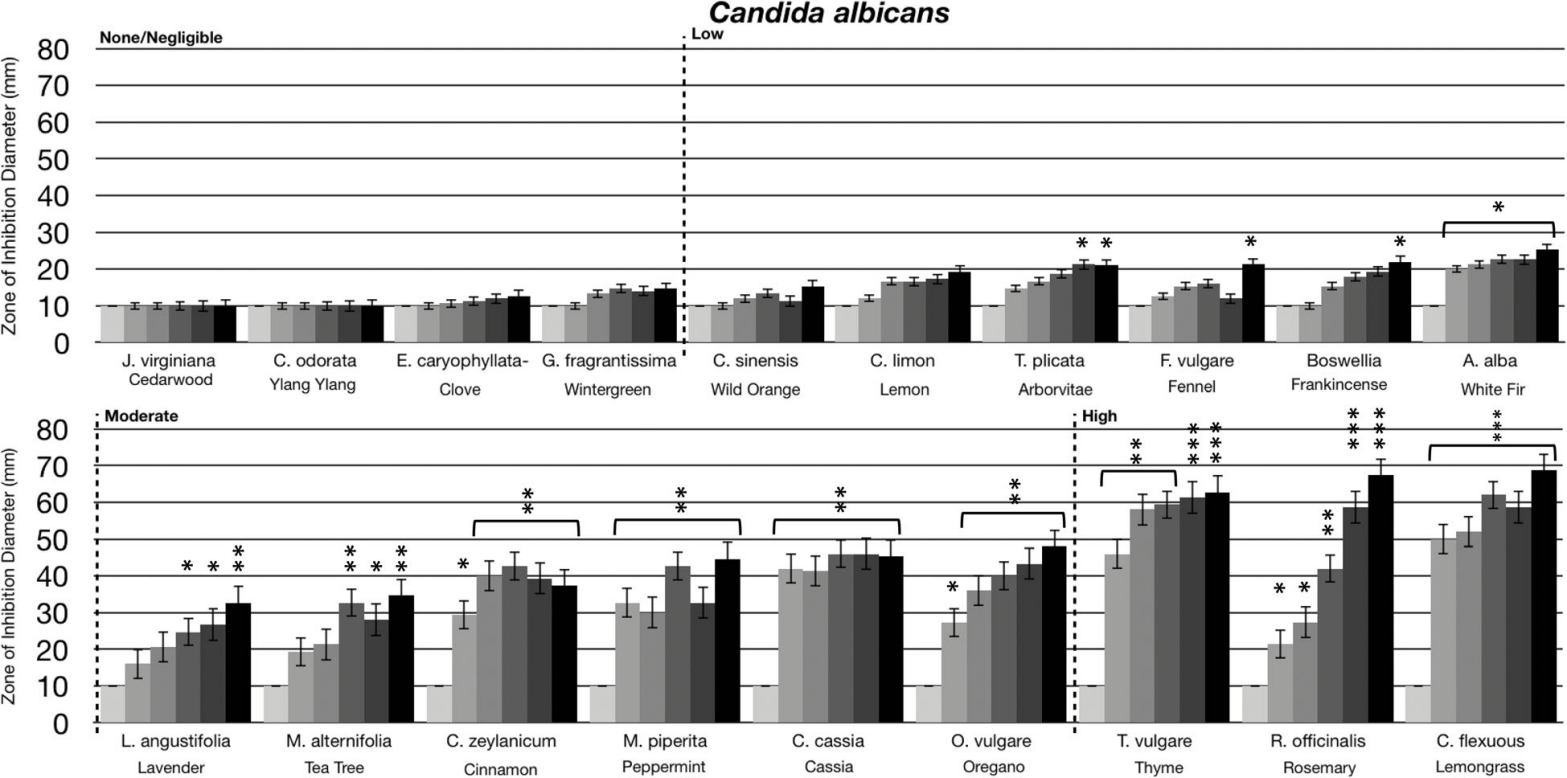
Anti-fungal activity of essential oil volatile constituents. Eighteen-hour broth cultures (*Candida albicans* (ATCC 10231)) were used to inoculate Sabouraud Dextrose Agar petri dishes (1 × 10 cfu/dish). A center plug of agar was removed and a sterile glass cylinder containing increasing amounts of essential oils (0 μL, 10 μL, 20 μL, 40 μL, 80 μL, 160 μL) was placed in the center of the petri dish. Petri dishes were incubated for 24 h at 37 °C. After 24 h of incubation, the zone of inhibition (diameter) was measured. The doses of 0 μL, 10 μL, 20 μL, 40 μL, 80 μL, 160 μL are shown on the graph from light grey to black, respectively. The antimicrobial activity of the essential oil volatiles was divided into six groups based on the zone of inhibition diameter: none (10 mm), negligible (10 mm - 15 mm), low (15 mm - 30 mm), moderate (30 mm - 50 mm), high (50 mm - 70 mm), and highest (70 mm - 80 mm). Error bars indicate the standard deviation from three separate trials. Statistical analysis was performed using a paired t-test. Statistically significant deviation of the various doses compared to untreated was indicated with asterisks: * *p* = 0.01–0.05; ** *p* = 0.001–0.01; ***p < 0.001

Statistical analysis was performed using a paired t-test. Statistically significant deviation of the various doses tested (10 μL, 20 μL, 40 μL, 80 μL, 160 μL) compared to untreated (0 μL) was indicated with asterisks in Figs. 1, 2, 3 and 4 with the *p*-value corresponding to the number of asterisks: * *p* = 0.01–0.05; ** *p* = 0.001–0.01; ***p < 0.001.

## Results

The antimicrobial activity of the volatile constituents from essential oils was divided into six groups based on the zone of inhibition diameter: none (10 mm), negligible (10 mm - 15 mm), low (15 mm - 30 mm), moderate (30 mm - 50 mm), high (50 mm - 70 mm), and highest (70 mm - 80 mm). Four Gram positive bacteria were tested including *S. epidermidis*, *S. pyogenes*, *S. aureus*, and *M. smegmatis*. As shown in Fig. 1a, *S. epidermidis* was not sensitive to clove, wild orange, ylang ylang, and fennel oils. Cedarwood, wintergreen, lavender, lemon, frankincense, cassia, peppermint, and cinnamon oils had low antibacterial activity. Oregano, white fir, arborvitae, tea tree, lemongrass, and rosemary oils had moderate antibacterial activity and thyme oil had high antibacterial activity (Fig. 1a). For *S. pyogenes*, cinnamon, lavender, white fir, cassia, and rosemary oils had high antibacterial activity, with lemongrass and thyme oils having the highest antibacterial activity (Fig. 1b). Notably, these two oils were highly active, even at the lowest dose where they almost completely cleared bacterial growth to the outer edge of the petri dish. For *S. aureus,* cedarwood, tea tree, rosemary, and thyme oils had moderate antibacterial activity, with none of the oils tested being classified as high or highest levels of activity against *S. aureus* (Fig. 1c). *M. smegmatis* was the most sensitive Gram positive bacteria to the oils. Lemon, cassia, fennel, arborvitae, cedarwood, cinnamon, and frankincense oils had moderate antibacterial activity, and nine of the 19 essential oils tested had a high or highest level of antibacterial activity against *M. smegmatis* (Fig. 1d). Of these, ylang ylang, oregano, lemongrass, tea tree, peppermint, and thyme oils had high antibacterial activity, while avender, white fir, and rosemary oils had the highest antibacterial activity (Fig. 1d). Overall, *M. smegmatis* and *S. pyogenes* were the most sensitive Gram positive bacteria to the volatile constituents from essential oils. The most potent evaporative essential oils which inhibited the Gram positive bacteria included thyme, rosemary and tea tree oils.

When comparing the antimicrobial activity of the volatile substances from oils against Gram negative and Gram positive bacteria, Gram positive bacteria were generally more sensitive to the evaporative constituents (compare Figs. 1 and 2). As shown in Fig. 2, three Gram negative bacteria were tested: *Pseudomonas aeruginosa, Bordetella bronchiseptica,* and *Klebsiella pneumoniae*. *P. aeruginosa* was very resistant to the antimicrobial activity of the essential oil volatiles with frankincense being the most effective with moderate antibacterial activity (Fig. 2a). *B. bronchiseptica* was the most sensitive Gram-negative bacteria tested with thyme, rosemary, cassia, and cinnamon oil volatile constituents having high antibacterial activity (Fig. 2b). *K. pneumoniae* was moderately sensitive to the volatiles with white fir, thyme, and tea tree oils having moderate antibacterial activity (Fig. 2c).

Antibiotic resistance is a significant problem in the healthcare industry. Therefore, we compared the activity of the essential oil evaporates against both Gram positive and Gram negative antibiotic sensitive and resistant bacterial strains, including *S. aureus* and *P. aeruginosa*. For *S. aureus*, the antimicrobial effects of the aerosolized evaporates were similar between the antibiotic sensitive and antibiotic resistant (methicillin resistant *S. aureus,* MRSA) strains (compare Fig. 3a to b). Lemon, white fir, and cinnamon volatile constituents had low antibacterial activity against the antibiotic sensitive *S. aureus* (Fig. 3a) while having moderate antibacterial activity against MRSA (Fig. 3b). Cedarwood, tea tree, thyme and rosemary volatiles had moderate antibacterial activity to both strains (Fig. 3a and b). Notably, as described by ATCC, this MRSA strain is resistant to a broad spectrum of antibiotic classes including ampicillin, amoxicillin/clavulanic acid, ciprofloxacin, cephalothin, doxycycline, gentamicin, erythromycin, imipenem, methicillin, penicillin, tetracycline, oxacillin, azithromycin, clindamycin, ceftriaxone, rifampin, amikacin and tobramycin. These results may suggest that the mechanism of action for MRSA resistance to these antibiotics does not provide resistance against the evaporative constituents present in lemon, white fir, cinnamon, cedarwood, tea tree, thyme and rosemary oils.

As was described in Fig. 2a, *P. aeruginosa* was not particularly sensitive to the volatile constituents in essential oils. When comparing the antibiotic resistant and sensitive strains of *P. aeruginosa*, most of the aromatic oils were still ineffective (compare Fig. 3c and d). However, frankincense oil, which had moderate activity against the antibiotic sensitive strain, did not have any activity against the antibiotic resistant strain (Fig. 3d). This may suggest that the mechanism of antibiotic resistance with this *P. aeruginosa* strain may also provide resistance to evaporative constituent(s) in frankincense oil.

Essential oils have been shown to have antimicrobial activity against fungi/yeast, including *Candida albicans*, when used in direct liquid contact [8, 9, 21, 22, 28, 29]. As with the antibacterial assays, we investigated the ability of essential oil evaporative constituents to inhibit the growth of *C. albicans*. As shown in Fig. 4, lavender, tea tree, cinnamon, peppermint, cassia, and oregano volatile constituents had moderate anti-fungal activity, with thyme, rosemary, and lemongrass having high anti-fungal activity. This supports that the active evaporative constituents present in some of these oils can not only inhibit prokaryotic growth, but eukaryotic (fungal/yeast) growth as well.

With the number of assays performed in this study, the activity of the essential oil volatile constituents could be compared and the most potent essentials oils identified. As shown in Table 1, cassia, tea tree, thyme, and rosemary volatiles had the broadest spectrum of antimicrobial activity, whereas clove, wild orange, ylang ylang, and fennel volatiles had the lowest level of activity. The other oils, including lavender, peppermint, lemon, cedarwood, frankincense, white fir, oregano, cinnamon, and lemongrass had notable antimicrobial activity, but it was more specific to either Gram positive bacteria or specific microbial species (Table 1).

**Table 1:**
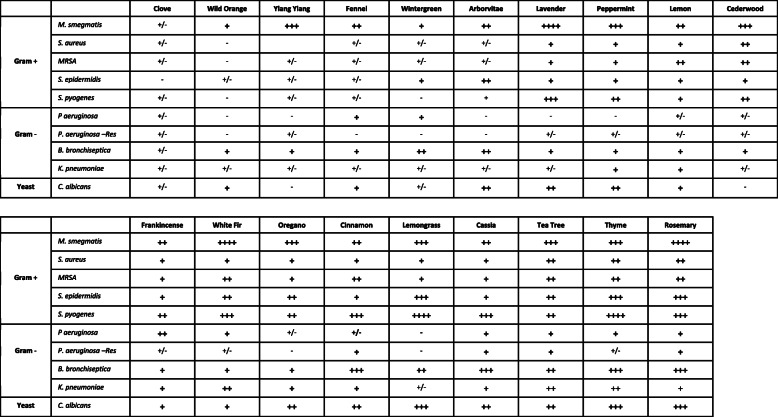
Summary of essential oil volatile constituent antimicrobial activity. The six divisions of antimicrobial activity are summarized with the following signs: - or +/− sign (none - negligible, 0 mm - 10 mm), + sign (low, 15 mm - 30 mm), ++ sign (moderate, 30 mm - 50 mm), +++ sign (high, 50 mm - 70 mm), and ++++ sign (highest, 70 mm - 80 mm). Each sign in the table represents the strength of the essential oil evaporative volatiles against the indicated microbe. The antimicrobial activity of the essential oil volatiles is graded from lowest on the left (clove), to highest on the right (rosemary)

## Discussion and conclusions

The goal of this study was to evaluate the antimicrobial activity of the volatile constituents released from essential oils. The results indicated that essential oils do in fact have antimicrobial activity in their airborne evaporative state. Rosemary, tea tree, and cassia volatiles were found to be the best broad-spectrum antibacterial agents. Other essential oils were found to have moderately broad antimicrobial activity including, from highest to lowest, thyme, cinnamon, oregano, white fir, and frankincense oils. For fungi, *C. albicans* was most sensitive to thyme and rosemary volatiles, further supporting their broad spectrum activity to include eukaryotic pathogens as well. Clove was the only essential oil tested that had zero or negligible antimicrobial activity in its evaporative state. Overall, rosemary and thyme were the most effective aromatic essential oils tested. Standards for testing antimicrobial activity of pharmaceutical drugs are codified by Clinical and Laboratory Standards Institute, CLSI [2, 30]. Antimicrobial susceptibility testing categories are similarly broken into diffusion and dilution assays. Sensitivity is categorized into three major groups of activity: resistant, intermediate, and susceptible. Antimicrobial susceptible disk assays have comparable results to the diffusion assay completed in this research study. Susceptible zone of inhibition diameters for effective antibiotics in diffusion assays against *Staphylococcus* species range from 14 to 29 mm using a 6 mm disk. Thirteen out of the 19 essential oils tested against *S. aureus* fell within this range, accounting for differences in the size of the glass cylinder and filter disk.

The results presented in this current study support the claims from previous studies using alternative methods to investigate the antimicrobial properties of airborne evaporative constituents of essential oils. For example, *Mycobacterium* and *Pseudomonas* have consistently been found to be sensitive and insensitive, respectively, to the volatile constituents in essential oils [4, 22]. *S. aureus* is a widely studied microorganism for which consistent results can be found when tested against specific essential oils. For instance, cinnamon, oregano, peppermint, lemongrass, and thyme oils are efficacious against *S. aureus* [22, 25, 31, 32]. Research may disagree on the degree of efficacy of certain essential oils, but their antimicrobial activity is undeniable. Continued research to understand the mechanism of action of essential oils against microbes, both through direct liquid contact and airborne evaporative exposure, will likely identify the specific chemical constituents responsible for the activity of essential oils that could be applied to standardize samples for the treatment of infectious disease.

Terpenes are the major chemical constituents that give essential oils their pleasant aroma and proposed therapeutic activity, whether through direct liquid interaction or through aerosolization [10]. The essential oils in this study demonstrated a variable range of anti-microbial activity against microbes. This suggests that the aerosolized evaporative constituents present in the different essential oils have a range of effects on microbial growth. It has previously been shown that crude essential oils have stronger antimicrobial activity as compared to the individual isolated constituents [33, 34]. As shown in Table 2, rosemary’s major chemical constituents were 1,8-cineole, α-pinene, and camphor. Individually, α-pinene and 1,8-cineole were previously found to have antimicrobial activity, but less than crude rosemary oil [34]. α-pinene was also found in fennel, but in lower concentrations, which is consistent with fennel’s lower antimicrobial activity (Table 2). Thyme’s major chemical constituents were thymol, para-cymene, γ-terpinene, and linalool (Table 2). Thymol is the most abundant constituent found in thyme and literature strongly supports thymol to be a strong antibacterial agent [33, 35, 36]. Terpinen-4-ol is the major chemical constituent found in tea tree essential oil [37]. This constituent’s antimicrobial activity can be augmented or reduced depending on the concentration of γ-terpinene. However, previous research has shown that the addition of γ-terpinene to thymol had no effect on antimicrobial activity [33]. In comparing cassia and cinnamon oils, both of these essential oils contained trans-cinnamaldehyde which is known to have antimicrobial activity [38]. The results demonstrated similar antimicrobial activity for cassia and cinnamon oils, however, cassia oil had a significantly higher percentage of trans-cinnamaldehyde (80% in cassia vs 35–56% in cinnamon). These variations in activity and chemical constituents support different and likely synergistic active constituents being responsible for the antimicrobial activity of these essential oils. Together, our results along with previous studies, support the antimicrobial activity of essential oils, but our results may suggest that aerosolization of these constituents in variable combinations may act together to achieve maximum efficacy.

**Table 2:**
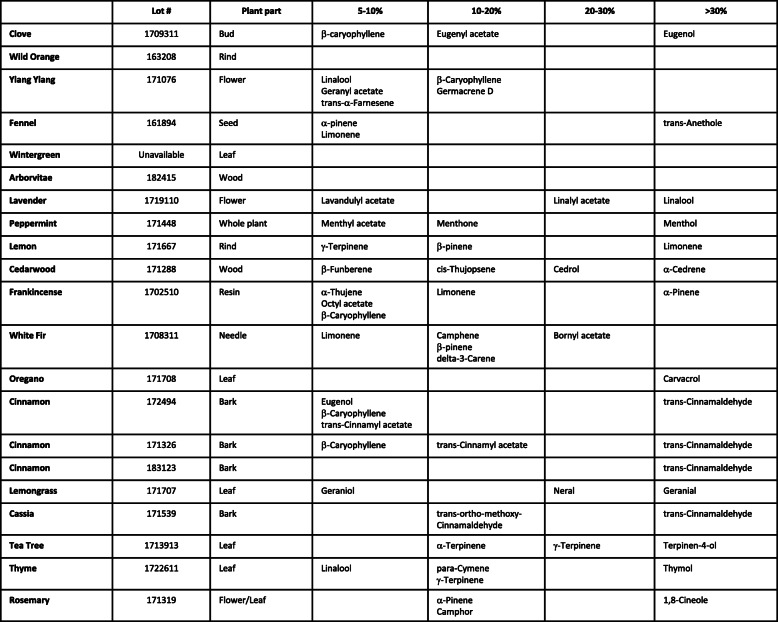
Constituents present in essential oils. Gas Chromatography/Mass Spectroscopy were completed for each essential oil by Essential Oil University. The chromatogram showed the percent area of each peak corresponding with each constituent. This table lists the chemical constituents present in each essential oil within the following ranges: 5–10%, 10–20%, 20–30, > 30%

In addition to the synergistic activity of essential oil constituents, the efficacy of the chemical constituents may differ depending on the method of application. For instance, clove showed limited antimicrobial activity in our volatile constituent assay. However, the literature supports clove and the major chemical constituent, eugenol, to be a strong antimicrobial in dilution assays where the oil is in direct liquid contact with the microbe [39–41]. In previous research, we tested cinnamon oil against *P. aeruginosa* in a dilution assay and found that cinnamon oil strongly inhibited *P. aeruginosa* when in direct liquid contact (data not shown) while our airborne evaporative assay showed only negligible antimicrobial activity. Thus, the antimicrobial activity of essential oils likely varies based on the use of airborne evaporatives or active constituents kept in a liquid form.

Natural products have historically been used to discover new chemical entities to treat infectious diseases [3]. Pathogens are increasing their resistance to current antibiotics. Essential oils provide a promising area of exploration to combat modern-day infections. When comparing the activity of the essential oils between antibiotic-sensitive and antibiotic-resistant *S. aureus* strains, similar levels of activity were observed. This suggests the mechanism that provides bacterial antibiotic resistance did not provide resistance to the essential oils. Conversely, antibiotic-sensitive *P. aeruginosa* was sensitive to the frankincense volatiles while the antibiotic-resistant strain was also resistant to the frankincense volatiles. This may suggest that the mechanism of antibiotic resistance for this strain of *P. aeruginosa* also provided resistance to the frankincense essential oil evaporatives.

*Mycobacterium smegmatis* is a bacterial strain related to the bacteria that causes the respiratory infection tuberculosis (*Mycobacterium tuberculosis*). This bacterium was found to be the most sensitive to the airborne evaporatives in essential oils. Interestingly, this bacterium is the only one that has a mycomembrane comprised of phenolic glycolipids [42]. This lipid-rich membrane allows the bacteria to be typically resistant to many drugs and has been related to its hyper-pathogenicity. The lipophilic nature of essential oils may be what allows these constituents to penetrate the lipid-rich membrane of *M. smegmatis*. Further investigation is warranted to explore the efficacy of essential oil volatile constituents against *M. tuberculosis*. With the rise of multi-drug resistant tuberculosis and extensive standard treatment protocols, investigation of other treatment options is imperative [43].

Potential future medical application of this research could be in the treatment of respiratory infections associated with *Mycobacterium*, *Klebsiella*, *Staphylococcus* and *Streptococcus* or dermatological infections from *S. aureus* and *C. albicans*. Potentially, essential oil evaporative diffusers could be modified to provide specific dosages of essential oil airborne volatiles at specified intervals in hospital rooms as an adjunctive treatment to reduce microbial load in respiratory or dermatological infections.

## Supplementary Information

**Additional file 1 Figure S1.** Example of glass cylinder evaporative zone of inhibition assay. As described in the Methods, bacterial or *Candida* broth cultures were used to inoculate agar petri dishes by spreading 1 × 10 colony forming units (cfu) in 100ul media on the surface of the agar. A 10 mm plug of agar was removed from the center of the petri dish and a sterile glass cylinder containing the indicated amounts of essential oil (0 μL, 10 μL, 20 μL, 40 μL, 80 μL, 160 μL) was placed in the hole in the center of the petri dish. The petri dishes were placed in a plastic container (to prevent any air flow currents from the incubator fan) and incubated for 24 h at 37 °C in an environmentally controlled incubator. After 24 h of incubation, the zone of inhibition (diameter) was measured.

## Data Availability

No additional information is supplied as a supplementary file. Additional questions or information may be obtained by contact the Corresponding author, Jeffrey Langland.

## Abbreviations

TSA: Tryptic Soy Agar
SDA: Sabouraud Dextrose Agar
