# Supplementary material for: Antimicrobial activity of the volatile substances from essential oils
Source: BMC Complement Med Ther. 2021 Apr 17;21:124. doi: 10.1186/s12906-021-03285-3 (PMC8053297; doi:10.1186/s12906-021-03285-3)
Supplement: Supplementary file 1 — Additional file 1 Figure S1. Example of glass cylinder evaporative zone of inhibition assay. As described in the Methods, bacterial or Candida broth cultures were used to inoculate agar petri dishes by spreading 1 × 10 colony forming units (cfu) in 100ul media on the surface of the agar. A 10 mm plug of agar was removed from the center of the petri dish and a sterile glass cylinder containing the indicated amounts of essential oil (0 μL, 10 μL, 20 μL, 40 μL, 80 μL, 160 μL) was placed in the hole in the center of the petri dish. The petri dishes were placed in a plastic container (to prevent any air flow currents from the incubator fan) and incubated for 24 h at 37 °C in an environmentally controlled incubator. After 24 h of incubation, the zone of inhibition (diameter) was measured. [file 12906_2021_3285_MOESM1_ESM.pdf]

Example of zone of inhibition assay using glass cylinders
(Staphylococcus aureus)

0 μL 10 μL 20 μL

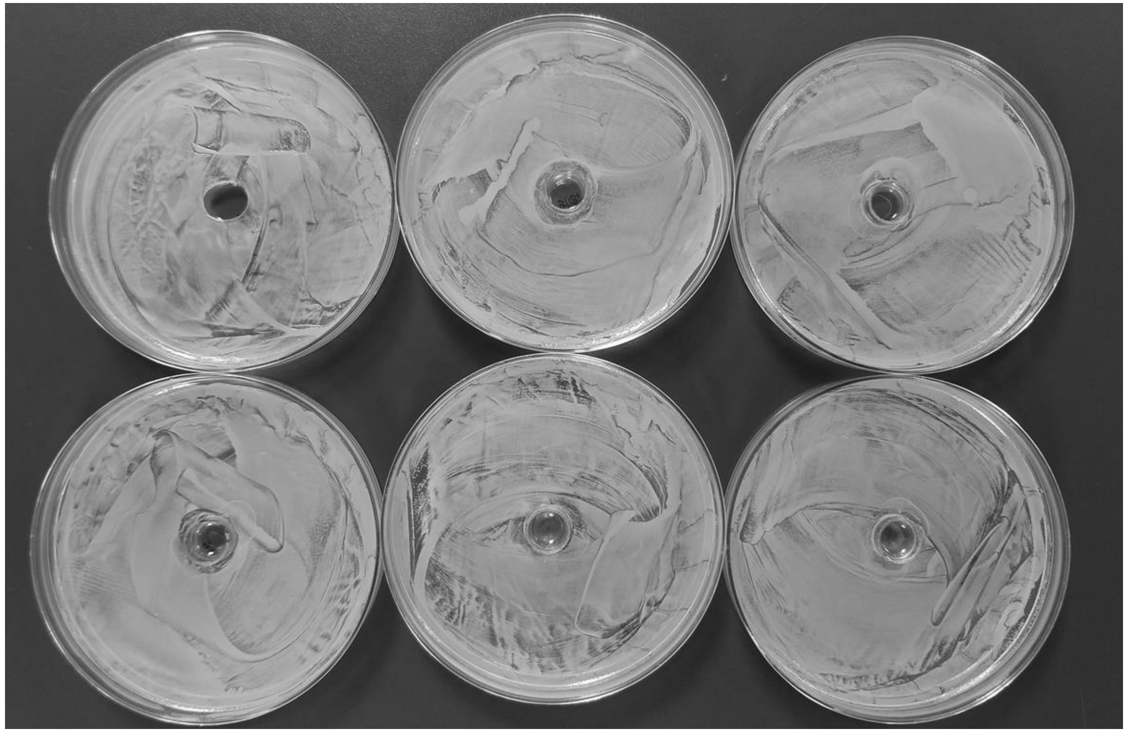

40 μL 80 μL 160 μL

Clove (*Eugenia caryophyllata*)

0 μL 10 μL 20 μL

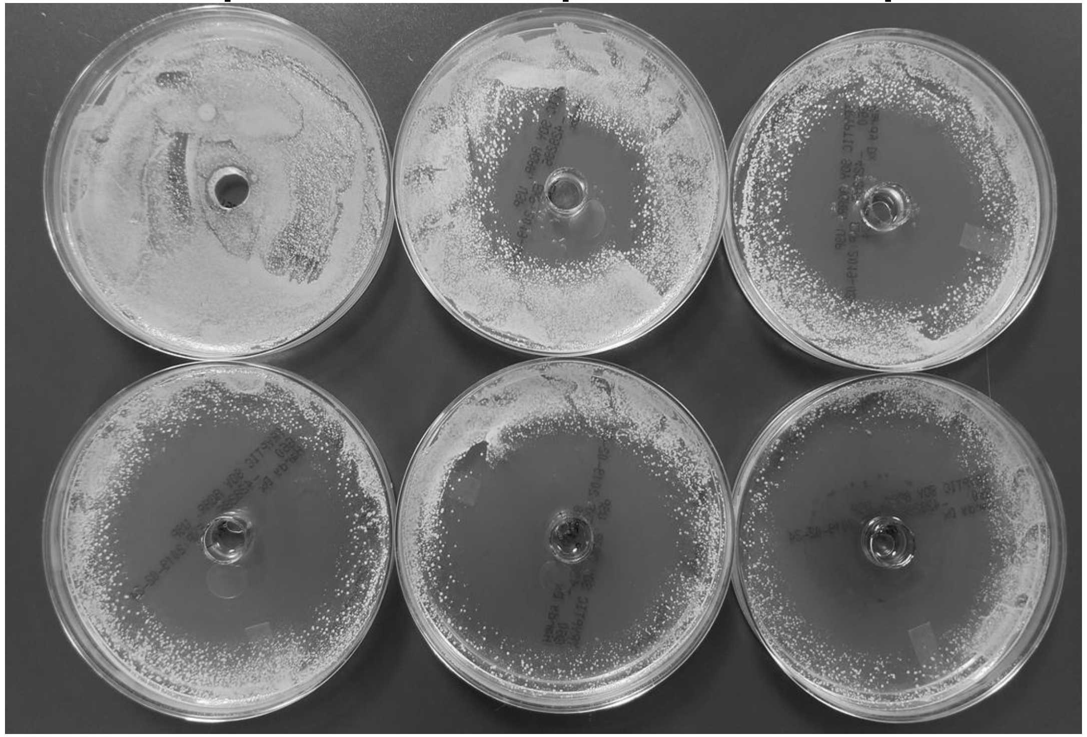

40 μL 80 μL 160 μL

Thyme (Thymus vulgaris)

Supplementary figure
